# Preoperative management of diabetes mellitus: A comparative narrative review of the recommendations of three professional organizations with Hamad Medical Corporation guidelines

**DOI:** 10.5339/qmj.2025.23

**Published:** 2025-02-04

**Authors:** Fathima Fahda M. Jamiu, Nudzejma Gostevcic, Yasser M. Hammad, Sohel M.G. Ahmed

**Affiliations:** ^1^Department of Anaesthesia, Intensive Care and Perioperative Medicine, Hamad Medical Corporation, Doha, Qatar; ^2^Department of Medical Education, Hamad Medical Corporation, Doha, Qatar; ^3^Weill Cornell Medicine-Qatar, Hamad Medical Corporation, Doha, Qatar; ^4^College of Medicine, Qatar University, Doha, Qatar *Correspondence: Sohel M.G. Ahmed. Email: sohelm@yahoo.com

**Keywords:** Diabetes mellitus, preoperative management, dipeptidyl peptidase-4 inhibitors, sodium–glucose cotransporter-2 inhibitors, glucagon-like peptide-1 agonists, guidelines

## Abstract

**Background:**

Diabetes mellitus (DM) is a prevalent condition that requires careful management in the perioperative setting to reduce surgical risks and optimize patient outcomes. The preoperative care of diabetic patients is complex because glucose control must be balanced with minimizing hypoglycemic or hyperglycemic events during surgery. A variety of diabetic medications such as insulin, dipeptidyl-peptidase-4 inhibitors, glucagon-like peptide-1 receptor agonists, sodium–glucose cotransporter-2 inhibitors, and biguanides such as metformin present unique challenges and considerations due to their different mechanisms, benefits, and potential adverse effects. In recent years, professional organizations have developed recommendations on the perioperative management of these therapies, although there remain some variations in guidelines related to medication cessation and dosage adjustments. Standardized protocols in preoperative DM care remain a topic of interest to ensure consistent and safe practices across healthcare settings, and further collaborative research efforts could provide clarity and consensus in managing this diverse patient population.

**Objective:**

The objective of this study was to provide an overview of guidelines and practices to support healthcare professionals in delivering improved preoperative care for diabetic patients. This initiative aims to enhance surgical outcomes and minimize the occurrence of complications.

**Methods:**

This is a comparative review that provides a systematic comparison of the similarities and differences in the guidelines and recommendations of three professional organizations along with Hamad Medical Corporation. All guidelines were from official websites including Hamad Medical Corporation.

**Conclusion:**

Although the reviewed guidelines for the preoperative care of diabetes patients share some similarities, there are also a number of differences due to outdated data and expert opinions, and therefore differ in practices around the world. While the key elements are agreed upon, more research and global collaboration are needed to create consistent guidelines and improve patient outcomes.

## Introduction

Diabetes mellitus (DM) represents a metabolic disorder characterized by inappropriately elevated blood glucose levels (BGL) resulting from either insulin deficiency or the body's inability to use insulin. DM characterized by insulin resistance is called type 2 DM (T2DM), while type 1 DM (T1DM) is characterized by complete insulin deficiency. tbl2DM is the predominant form globally and is becoming more common due to changes in lifestyle and longer life expectancy.^1^

Recent studies suggest that approximately 537 million individuals worldwide have diabetes. The number is expected to reach 700 million by the end of 2024.^2^ According to the IDF (International Diabetes Federation) report for 2021, Qatar ranks 14th globally with a diabetes prevalence rate of 19.5%. This means that one in six people in Qatar are diagnosed with diabetes. The aim of a study conducted by WCM-Q (Weill Cornell Medicine in Qatar) was to determine the prevalence and risk factors associated with DM in Qatari nationals. The study found that the incidence of tbl2DM is expected to double from 7% to 14% between 2021 and 2050.^3^

Several studies have highlighted an increased likelihood of complications in individuals with diabetes.^2,4,5^ The increased risk could be due to the seriousness of the condition that requires surgery, or hyperglycemia itself may contribute to complications in the postoperative period. Surgery, as well as anesthesia, and illness trigger the release of hormones such as cortisol, glucagon, growth hormone, and catecholamines due to the stress they induce. This process results in insulin production, insulin resistance, decreased peripheral tissue glucose utilization, increased lipolysis, and increased proteolysis. Consequently, there is an increase in gluconeogenesis and glycogenolysis, resulting in exacerbated hyperglycemia known as stress hyperglycemia.^4^

Interestingly, hyperglycemia also occurs frequently in individuals without DM during the perioperative period. Observational studies suggest that hyperglycemia in the preoperative period, regardless of the patient's diabetes status, can be used as a predictor of different postoperative complications such as infections, thromboembolic events, cardiovascular (CVS) complications, renal insufficiency, or prolonged hospital stay. Uncontrolled BGL cause osmotic diuresis, resulting in electrolyte imbalances as well as ketone formation and release of inflammatory cytokines. This ultimately results in damage to cell structures such as mitochondria, as well as impaired function of endothelial cells and disruption of immune system control. Therefore, maintaining optimal glucose control during the perioperative period is associated with favorable postoperative outcomes.^4^

Despite this evidence, there is a lack of research studies investigating the effectiveness of managing BGL at the time of surgery to reduce the risk of complications.^4^

There are different recommendations for the preoperative management of DM, based on expert opinion and consensus from best practice panels. This has occasionally led to variations in the clinical practices of different medical centers worldwide. The inconsistencies in these recommendations highlight both the limited evidence available for managing patients in the preoperative period and the reliance on outdated recommendations. The objective of this narrative review is to compare the similarities, differences, and suggestions of four professional bodies, namely the Hamad Medical Corporation in Qatar (HMC-Qatar), the Association of Anaesthetists (AoA), formerly known as the Association of Anaesthetists of Great Britain and Ireland (AAGBI), American Diabetes Association (ADA), and the Australian and New Zealand College of Anaesthetist guidelines in collaboration with the Australian Diabetes Society (ADS–NZCA), and to draw comparisons between them.

## Diabetes Mellitus

While tbl1DM and tbl2DM are primary types of DM, further classification includes MODY (Maturity-Onset Diabetes of the Young), pregnancy-related diabetes (also known as gestational diabetes), neonatal diabetes and secondary form related to endocrine disorders, steroid use and various other factors.

T1DM typically affects younger individuals or adolescents and is characterized by the destruction of pancreatic beta cells, often due to an autoimmune reaction. The result is a lack or reduced availability of beta cells, and therefore an insufficient or severely limited supply of insulin. The diagnosis of tbl1DM is usually made based on specific symptoms such as increased urination, excessive hunger, as well as extreme thirst and BGL (fasting glucose above 126 mg/dL, random glucose above 200 mg/dL, or hemoglobin A1c (HbA1c) above 6.5%) with or without the presence of antibodies against glutamic acid decarboxylase and insulin.^6^

T2DM often occurs in middle-aged adults whose BGL is elevated due to inactive lifestyles and poor eating habits. It is characterized by a gradual imbalance between insulin levels and the body's response to them, leading to reduced hormonal function. Insulin resistance is a complex illness that often arises from the combined effects of obesity and the biological aging process. It is crucial to assess fasting glucose levels and conduct testing promptly for diagnosing tbl2DM. Individuals with borderline glucose levels may need an oral glucose tolerance test (OGTT) for evaluation. The OGTT evaluates both fasting glucose levels and the body's response to a glucose load for diagnosing tbl2DM.^6^

Moreover, according to the ADA guidelines, diabetes can be diagnosed using any of the following criteria:

i. A level of 6.5% or greater ( = 48 mmol/mol).

ii. Fasting plasma glucose level of 126 mg/dL ( = 7.0 mmol/L) or greater, after eight hours of fasting before calorie intake.

iii. Two hours, after taking an OGTT with 75 grams of glucose diluted in 250–300 mL of water.

iv. A plasma glucose level of 200 mg/dL ( = 11.1 mmol/L) or greater indicates hyperglycemia symptoms such as increased thirst, appetite, or weight loss.^7^

## Antidiabetic Medications

Due to the increasing prevalence of DM patients globally, new and advanced classes of medications have been developed to address this growing concern. The different types of diabetic medications commonly prescribed for managing DM are typically classified based on their pharmacological effects:

### Insulin

Insulin is a type of antidiabetic medication used to treat both forms of DM. Its mode of action is demonstrated by binding to its receptors on the plasma membranes of cells. Insulin drugs are classified according to their duration of action: rapid-acting insulins (lispro and aspart), short-acting insulins (regular insulin), intermediate-acting insulin (NPH – Neutral Protamine Hagedorn), and long-acting insulins (glargine, detemir). The route of administration can be subcutaneous (SC), intravenous (IV), or intramuscular (IM), depending on the patient's health status and clinical circumstances.^8^

Side effects are divided into two categories: those caused by the drug and those caused by the method of administration. The most common side effects include hypoglycemia and weight gain. In rare cases, insulin can cause electrolyte imbalances such as hypokalemia. When discussing the side effects of insulin, it is crucial to mention two occurrences: the Somogyi effect and the dawn phenomenon. The Somogyi effect occurs in certain individuals in the early morning and manifests itself as rebound hyperglycemia due to the action of insulin on the body, resulting in a hypoglycemic condition. In this case, two hormones, cortisol and adrenaline, play a significant role in generating this negative effect.^8^

The dawn phenomenon, on the contrary, is characterized by excessively high BGL in the early morning, which is due to inadequate insulin dosage before the occurrence of this phenomenon.^8^

Furthermore, insulin intoxication may manifest itself as very low BGL, which causes symptoms such as nausea, palpitations, sweating, abdominal pain, and impaired vision, requiring immediate medical attention.^8^

### Biguanides

Metformin, the most commonly used biguanide for managing DM, is an oral antihyperglycemic drug approved by the US Food and Drug Administration (FDA) in 1994 for treating tbl2DM. It is considered the first choice for monotherapy in diabetes treatment.^9,10^ Metformin functions by activating the AMPK (Adenosine Monophosphate-activated Protein Kinase) enzyme, which plays a role in regulating genes related to hepatic gluconeogenesis, thereby decreasing hepatic glucose production, reducing intestinal absorption, and enhancing insulin sensitivity.^11^ In general, metformin is considered safe and well tolerated. However, some gastrointestinal (GI) side effects such as nausea, vomiting, and diarrhea can limit its use.^9,10^ Furthermore, metformin should not be used in patients with severe renal failure, medication hypersensitivity, or metabolic acidosis.^10^

### Sulfonylureas

Sulfonylureas, such as gliclazide, glibenclamide, and glipizide, have been used as second-generation medications for DM treatment since the 1950s. They are the oldest type of antidiabetic medications available.^11,12^ Glimepiride, which came onto the market in 1995, is a relatively newer drug compared to medications in the same class. This drug has a lower affinity for cardiac muscles and is not associated with CVS side effects compared to other sulfonylureas. These medications are popular due to their affordability. Their function is to increase endogenous insulin production through a process that blocks ATP-sensitive potassium (K^+^) channels on pancreatic beta cells, reducing K^+^ efflux and causing depolarization of the cell membrane. Once the membrane depolarizes, the calcium (Ca^2+^) channels open and cause an influx of Ca^2+^, leading to insulin production regardless of BGL.^12^ One of the common side effects associated with these medications is hypoglycemia. Other negative outcomes include weight gain, nausea, dizziness, migraines, and facial flushing. Sulfonylureas should be avoided in elderly patients due to the risk of hypoglycemia and in individuals with liver or renal impairment.^12^

### Thiazolidinediones (TZDs)

TZDs, such as rosiglitazone and pioglitazone, are a type of oral hypoglycemic agents (OHAs) that improve insulin sensitivity by activating the nuclear transcription factor PPAR-γ (Peroxisome Proliferator-Activated Receptor-Gamma) in liver hepatocytes and cardiac muscles. The primary side effect of TZDs is weight gain. Less common side effects may include edema, congestive heart failure, bone fractures, bladder cancer, hepatotoxicity, diabetic macular edema, increased ovulation, and teratogenic effects.^13^

TZDs can be used as monotherapy because they do not cause hypoglycemia and are safe for patients with chronic kidney disease (CKD), which is the main advantage of using this drug. However, they should be avoided in individuals with heart failure, moderate to severe hepatic failure, bladder malignancy, pregnancy, or in those at risk of bone fractures (osteoporosis).^13^

### Dipeptidyl peptidase-4 inhibitors (DPP4-i)

DPP4-i, also known as gliptins, include sitagliptin, vildagliptin, saxagliptin, alogliptin, and linagliptin. These medications function by blocking the DPP4 enzyme, leading to increased levels of glucagon-like peptide-1 (GLP-1) and gastric inhibitory polypeptide. This process triggers pancreatic beta cells to produce insulin and GLP-1. Additionally, DPP4-i reduce glucagon production by pancreatic alpha cells. Patients with tbl2DM can effectively manage their BGL through these combined effects. Overall, DPP4-i is well tolerated and has fewer adverse effects.^9,11^

### Glucagon-like peptide-1 agonists

GLP-1 medications are commonly used for treating tbl2DM and obesity. Drugs in this class include exenatide, lixisenatide, liraglutide, albiglutide, dulaglutide, and semaglutide, with the latter being the most commonly used.^14^ GLP-1 agonists have gained popular due to their ability to reduce HbA1c levels and promote weight loss in obese individuals.^9,14^ They are considered safe for patients with certain medical conditions such as heart failure, CKD, and atherosclerosis.^14^ These drugs are incretin-based, which increases insulin production, decreases glucagon secretion, and reduces hepatic glucose production.^9^ The typical adverse effects associated with GLP-1 agonists mostly involve problems related to the GI tract such as nausea, vomiting, diarrhea, and dyspepsia. Additionally, they are known to delay gastric emptying, which has led to increased concerns about the risk of aspiration.^15^ Other side effects may include headache, dizziness, a slight increase in heart rate (tachycardia), and increased satiety. The use of GLP-1 agonists is discouraged in patients with hypersensitivity to the drug, in pregnant individuals, and in patients with gastroparesis or inflammatory bowel disease. Patients with a family history of multiple endocrine neoplasia 2A or 2B and medullary thyroid cancer should also avoid these medications.^14^

### Sodium–glucose cotransporter-2 inhibitors (SGLT-2i)

SGLT-2i is a relatively new class of drugs used for diabetes treatment. These medications function by blocking SGLT-2 receptors in the proximal tubules of the kidney, leading to increased urinary glucose excretion. Consequently, this action helps reduce BGL.^16^ SGLT-2i drugs, also called gliflozins, include dapagliflozin, empagliflozin, and canagliflozin, which have the added benefit of reducing HbA1c levels while reducing body weight and normalizing blood pressure.^9,16^ Some research studies have indicated that these medications also have beneficial effects on the heart and kidneys and are therefore useful for patients with heart failure and CKD.^16^ They are generally well tolerated, with some side effects such as genital fungal infections, urinary tract infections, and increased urination, with the incidence being higher among women.^9,11,16^ A serious side effect of this class of drugs is the risk of diabetic ketoacidosis (DKA), particularly during the perioperative period due to prolonged fasting while taking this medication.^16^

## Use Of Hba1c In The Preoperative Setting

Various guidelines emphasize the importance of monitoring HbA1c levels as an indicator of DM control in the context of preoperative management. The updated 2024 clinical protocol at HMC-Qatar suggests that for elective surgical procedures, HbA1c levels should be ≤ 8.5% ( = 69 mmol/mol). If it is not possible to achieve an HbA1c level ≤ 8.5% ( = 69 mmol/mol), the preoperative BGL should be maintained between 5.6 and 12 mmol/L. Patients with HbA1c levels above 8.5% ( ≥ 69 mmol/mol) are advised to be admitted one to two days before surgery to optimize BGL. This proactive approach prioritizes control of BGL before surgical procedures to improve overall outcomes.^17^

The 2015 AoA guidelines suggest that if the HbA1c level is above 8.5% ( ≥ 69 mmol/mol), referral should be considered for optimization. Moreover, they suggest delaying surgery if the HbA1c level is unexpectedly 8.5% ( ≥ 69 mmol/mol). These guidelines underscore the importance of achieving glycemic control before elective surgery and emphasize the consensus on various recommendations for careful management of HbA1c levels to improve perioperative outcomes.^18^

The ADA focuses on elective procedures and recommends that the HbA1c level should ideally be below 8% ( < 64 mmol/L). This highlights the importance of regulating BGL before surgery in individuals with diabetes to optimize surgical outcomes.^19^

The ADS–ANZCA highlights the significance of considering HbA1c levels when planning elective surgical procedures. For individuals diagnosed with DM and have a high HbA1c level of 9% or greater ( ≥ 75 mmol/mol), it is recommended that delaying surgery be considered due to evidence of poorer outcomes. It is recommended to assess glycemic stability and consider postponing surgery if the patient is not in optimal condition on the day of the procedure. The guidelines acknowledge that improving glycemic control may require adjustments to current diabetes treatment, potentially leading to a delay in surgical procedures of more than three months in certain situations.^20^

In summary, although there are some differences in exact numerical targets, these guidelines collectively highlight the significance of HbA1c levels during preoperative assessments (Table 1). Their advocacy involves implementing proactive management strategies such as including admission for optimization, delaying surgery when HbA1c levels are high, and using tailored glycemic control measures to improve perioperative outcomes. All of these recommendations from various guidelines demonstrate the intricate relationship between the management of diabetes and the problems associated with surgery.

## Insulin-like Antidiabetic Medications In The Preoperative Setting

The HMC-Qatar guidelines clearly outline insulin administration in various circumstances during the preoperative period. Patients transitioning from DM management through diet with fasting blood glucose (FBG) levels between 10 and 13.9 mmol/L should initiate glucose–insulin–potassium (GIK) therapy along with additional bolus insulin in the form of rapid-acting antihyperglycemic agent (aspart or lispro). Insulin is administered subcutaneously in its basal form, similar to long-acting agents (glargine), at a concentration of 0.2 units per kilogram on the morning of surgery to patients who have previously taken OHAs or incretins.^17^

In individuals who have previously received insulin therapy, the total daily SC insulin dose should be reduced by 20–30%. The basal insulin dose can be administered before surgery even if the patient has been fasting since midnight.^16^ Additionally, according to the HMC-Qatar guidelines, it is recommended to reduce the basal infusion rate by 20–30% in patients who previously used an insulin pump and had controlled diabetes. However, switching from an insulin pump to basal–bolus insulin may be necessary if the patient is undergoing surgery, is critically ill, or has no trained provider available.^17^

If patients are hospitalized for more than 48 hours and are using insulin, they should be switched to SC basal–bolus insulin. Half of the basal insulin dose (glargine) is administered once daily at bedtime and the other half is administered in three separate doses with meals as bolus insulin (aspart or lispro).^17^

The HMC-Qatar guidelines recommend administration of two-thirds of the usual morning dose as basal insulin SC glargine if the patient has previously received intermediate-acting NPH insulin once daily in the morning. However, if the patient is receiving premixed (short-acting and long-acting) insulin, glargine should be administered subcutaneously at half the usual dose on the morning of surgery.^16,17^

The HMC-Qatar guidelines recommend not taking prandial insulin (regular lispro, aspart, and glulisine) during fasting. Additionally, basal insulin administration should be discontinued on the day of surgery and resumed at two-thirds of the dose in the evening or early morning as mentioned previously.^17^ However, if the FBG falls within the range of 10–13.9 mmol/L, GIK therapy can be initiated with the addition of a short-acting medication and the diabetes team should be consulted.^17^ Furthermore, the HMC-Qatar guidelines emphasize the measurement of the capillary blood glucose every 4 hours preoperatively while on IV fluids and supplements with the bolus insulin regimen subcutaneously.^17^

According to the AoA guidelines, it is recommended to reduce the dosage of long-acting insulin agents by 20% the day before surgery while closely monitoring BGL and administer the remaining 80% of the dose. When using a combination of short-acting, rapid-acting, and intermediate-acting insulin (biphasic) or ultra-long-acting insulin, it is recommended to administer half of the morning dose while keeping the evening dose constant.^20^

In the standard regimen, the total dose of morning insulin should be determined when short-acting and intermediate-acting insulin are administered separately through two injections per day. Half of this calculated dose should be given as intermediate-acting insulin in the morning, while the evening dose should remain constant.^20^

If a patient typically takes 3–5 daily doses of insulin, they should adjust their basal–bolus routine by omitting the morning and lunchtime doses while maintaining basal insulin levels. In the case of a morning surgical procedure, reduce the morning dose of mixed insulin by half and skip the midday dose. However, if the surgery is scheduled for the afternoon, stick with morning doses while skipping insulin injections.^20^

Although there are no specific guidelines from the ADA regarding the preoperative management of patients, it is essential to note that the ADA emphasizes the IV administration of insulin in critical care settings to maintain optimal glycemic control. However, in non-critical care situations, it is advisable to administer SC rapid or short-acting insulin before meals or every 4–6 hours when the patient is fasting or receiving continuous enteral or parenteral nutrition.^19^

The ADS–ANZCA guidelines recommend that patients undergoing surgical procedures provide clear written instructions for their insulin regimen at home before visiting the hospital. It is recommended that the insulin regimen be continued up to and including the night before the procedure. Furthermore, for basal insulin regimen, it is generally recommended to maintain the dose and timing unless episodes of overnight hypoglycemia have occurred. In this case, the dosage should be reduced by 20%. However, if a combination of premixed insulin is used, it is recommended to reduce the morning dose by half before surgery and omit the lunchtime dose.^11^ If the patient has previously received co-formulated insulin (ultra-long-acting basal insulin analogue and rapid-acting insulin combination) and the procedure is scheduled for the morning hours, the morning dose should be delayed until lunchtime (unless fasting). Nevertheless, if the treatment is scheduled for the afternoon, the morning dosage can still be administered, although at a 50% lower amount, and skipped during lunchtime. If the patient maintains regular food and drink intake, the evening dosage can be administered as usual. The ADS–ANZCA guidelines emphasize the significance of careful blood glucose monitoring, starting from the moment the patient wakes up at home and continuing at intervals of 1–2 hours until arrival at hospital. The ADS–ANZCA recommends a BGL target of 5–10 mmol/L.^11^

Additionally, these guidelines may vary slightly between general surgical procedures and day procedures such as colonoscopy and gastroscopy that involve bowel preparation (Table 2). Patients undergoing day procedures such as colonoscopy can continue their basal insulin regimen. However, it is possible to administer intermediate-acting, premixed, and co-formulated insulin in the morning while excluding it during lunchtime. Short-acting insulin is administered exclusively for afternoon procedures, and the patient should consume a small breakfast.^11^

## Use Of Variable Rate Intravenous Insulin Infusion (Vriii) In The Preoperative Setting

The HMC-Qatar recommendations suggest opting for VRIII over GIK in scenarios such as surgical procedures lasting more than 2 hours in the ICU (Intensive Care Unit) patients, emergency surgical cases, or situations where BGL cannot be effectively managed with GIK. The HMC-Qatar guidelines highlighted the importance of daily monitoring of serum electrolytes while the patient is on VRIII and recommended continuous use of VRIII (unless basal insulin is administered) if the patient has tbl1DM to reduce the risk of hospital-acquired DKA.^16^

The AoA guidelines recommend the use of VRIII for patients who are likely to miss multiple meals, patients with tbl1DM undergoing surgery without receiving background insulin, uncontrolled diabetes, or emergency surgical procedures.^20^

However, the ADA guidelines have no specific recommendations regarding the use of VRIII in preoperative circumstances. In contrast, the ADS–ANCZA guidelines advocate the use of VRIII only during the intraoperative phase and in cases of rapid increase in BGL (Table 3).^11,19^

## Metformin In The Preoperative Setting

The HMC-Qatar guidelines recommend discontinuing biguanides (metformin) on the day of surgery and discontinuing them for 24 hours before any procedure in patients above 65 years of age or those with liver disease, heart failure, or renal problems. The guidelines emphasize the need to stop taking biguanides for 48 hours after surgery if IV contrast is used, and then continue the medication once the patient is stable, eating, passing urine normally, and their renal function is within the baseline levels.^17^

According to the AoA guidelines, patients with morning or afternoon procedures should maintain their metformin dose until the day before admission unless they need IV contrast. In this case, it is recommended to discontinue the medication until they can resume eating and drinking normally. These instructions also apply to patients undergoing procedures without contrast media. Additionally, individuals with pre-existing kidney problems or at risk of kidney damage should discontinue biguanide due to the risk of acidosis from high levels of the drug in the bloodstream since metformin is excreted through the kidneys.^20^

The ADA guidelines propose an approach in which metformin is discontinued on the day of surgery and resumed once eating normally, as well as when renal function returns to baseline within 24–48 hours after surgery.^19^

The ADS–ANZCA guidelines recommend discontinuing the use of metformin in patients with impaired kidney function (CKD stage 3B or lower/estimated Glomerular Filtration Rate (eGFR) < 45 mL/min/1.73 m^2^), elevated BGL on admission (BGL >12 mmol/L), and for specific medical procedures such as colonoscopy or major surgery. Metformin can be temporarily stopped for day operations and immediately resumed once the patient resumes oral intake. Metformin should not be resumed for at least two days after major surgery and only until the patient has fully resumed a normal diet.^11^ A summary of the metformin management in the preoperative setting is shown in Table 4.

## Sodium–glucose Cotransporter-2 Inhibitors In The Preoperative Setting

The HMC-Qatar guidelines recommend a cautious approach to managing patients taking SGLT-2i (such as dapagliflozin, empagliflozin, and ertugliflozin) before surgery. According to these guidelines, SGLT-2i drugs should be stopped three days before the scheduled elective surgery. Medications can be restarted as soon as the patient awakens and eats after surgery.^17^

Additionally, the AoA's 2015 guidelines updated in 2019 warns against DKA during fasting and recommends stopping SGLT-2i on the day of surgery. Patients are advised to continue taking their medications as usual on the day before surgery. Additionally, if the patient is using a VRIII, it is recommended that use of SGLT-2I be omitted until the patient resumes normal eating and drinking habits.^18^

The ADA and US FDA guidelines suggest a withdrawal period of three to four days before surgery to mitigate the risk of DKA related to these medications during the phase.^16,19^

Conversely, the ADS–ANZCA guidelines propose an alternative approach to managing SGLT-2i before surgery. According to their guidelines, SGLT-2i should be withheld for two days before surgery and on the day of surgery. However, an exception applies to patients having minor surgery, including day-procedure patients who only need to omit their SGLT-2i on the morning of their procedure. This comprehensive technique takes into account the specific type and severity of the surgical procedure.^11^

In summary, although all guidelines emphasize stopping SGLT-2i in the preoperative period to prevent complications, there are differences in the recommended duration of cessation and specific considerations for the type of surgery across these diverse guidelines (Table 5). The discrepancies indicate the need for a careful and subtle strategy to maintain glycemic control and reduce potential hazards in the perioperative care of patients on SGLT-2i inhibitors.

## Glucagon-like Peptide-1 Receptor Agonists In The Preoperative Setting

The HMC-Qatar, the ADA, and the ADS–ANZCA guidelines have a commonality when it comes to GLP-1 receptor agonists, which include medications such as exenatide, liraglutide, dulaglutide, and semaglutide in their respective recommendations for the preoperative management of diabetic patients. All three bodies recommend continuing GLP-1 medications until the night before surgery and on the morning of surgery.^11,17,19^

The AoA guidelines provide a different perspective on GLP-1 receptor agonists in the preoperative setting. For patients undergoing surgery in the morning or afternoon, it is recommended that these medications be taken as usual until surgery, even if the patient is fasting. It is important to continue the use of GLP-1 receptor agonists even if a VRIII is administered. This pragmatic approach emphasizes the complex nature of preoperative care and aims to achieve an appropriate balance between medication administration and the unique requirements of each patient.^18^

As previously mentioned, GLP-1 medications are considered important for their effectiveness in DM treatment and weight loss. However, the anesthesia community worldwide has raised significant concerns about these treatments because they are likely to be associated with a higher risk of aspiration. The main cause for alarm is that they tend to delay stomach emptying when taken over a long period of time. This poses a major difficulty for anesthesiologists, who must closely monitor these risks during procedures involving patients on GLP-1 therapy and adopt specific strategies to minimize them.^15^ According to the guidelines of the American Association of Anesthesiologists, it is recommended that GLP-1 drugs be stopped on the day before surgery. Patients taking weekly GLP-1 medications should discontinue them one week before surgery.^21^

In summary, the HMC-Qatar, ADS–ANZCA, and ADA guidelines recommend discontinuing GLP-1 receptor agonists on the day of surgery. However, the AoA guidelines provide a more adaptable approach by allowing their use in certain circumstances. The variations in recommendations emphasize the need for a tailored and patient-centered approach to managing GLP-1 receptor agonists in the perioperative context (Table 6).

## Dipeptidyl-peptidase-4 Inhibitors (Dpp4-i) In The Preoperative Setting

The guidelines for the preoperative management of DM related to DPP4-i, including sitagliptin, vildagliptin, saxagliptin, linagliptin, and alogliptin, vary in their recommendations. The HMC-Qatar, ADA, and ADS–ANZCA guidelines suggest discontinuing DPP4-i on the morning of surgery if the patient is NPO (Nil Per Os), while the last dose should be taken the night before surgery. This drug is included in the category of all OHAs in all three guidelines. This strategy is consistent with a careful approach to non-insulin medications in the immediate perioperative period.^11,17,19^

The 2019 AoA guidelines adopt a more liberal approach, indicating that DPP4-i can be maintained during periods of fasting. Patients scheduled for morning or afternoon procedures should continue to take DPP4-i medications as prescribed. However, if VRIII is administered, these drugs should be stopped until the patient is eating and drinking normally.^18^

In summary, the guidelines of the four professional bodies show variations in their recommendations for DPP4-i in the preoperative management of DM (Table 7). While several guidelines recommend maintaining the regular use of the medication until the day of surgery, others propose stopping it on the morning before surgery. This reflects the importance of tailoring the strategy to each patient's specific characteristics and surgical factors.

## Other Oral Hypoglycemic Agents In Preoperative Settings

According to the HMC-Qatar guidelines, it is recommended to discontinue the use of agents such as sulfonylurea, TZDs, and α-glucosidase inhibitors if a patient is NPO before surgery. Further management focuses on the patient's HbA1c level from the laboratory work-up before admission. If HbA1c is >8% (>64 mmol/mol), administer 0.15–0.3 units/kg SC glargine in addition to GIK, D5% normal saline (NS), or half NS on the day of surgery. If the patient's FBG is < 10 mmol/L, it is recommended to administer IV fluid D5% NS or D5% half NS if NPO preoperatively for more than 8 hours. However, if the range is between 10 and 13.9 mmol/L, GIK with correctional aspart or lispro should be initiated subcutaneously. An FBG level exceeding or equal to 14 mmol/L requires the diabetes team to be involved according to the HMC-Qatar guidelines.^17^

The AoA guidelines provide clear instructions on the use of medications such as meglitinides, sulfonylurea, and acarbose, which should be administered as usual one day before admission.^19^

If the patient's surgical procedure is scheduled for the morning, it is recommended to skip the morning dose of meglitinides and acarbose. Nevertheless, the morning dose should be administered as usual if the surgery is scheduled for the afternoon of the same day. However, they recommended discontinuing sulfonylurea on the day of surgery, regardless of the timing of surgery. If the initiation of VRIII occurs, it is necessary to discontinue the administration of meglitinides and acarbose until the patient resumes regular food and beverage intake.^18^

According to the ADA guidelines, it is recommended that any additional OHA be discontinued on the morning before surgery or procedure. Instead, it is recommended that patients receive a half dose of NPH insulin or 75–80% of long-acting analogue insulin or insulin pump, basal insulin, according to the type of DM and clinical judgment.^19^

According to the ADS–ANZCA guidelines, it is recommended that all non-insulin antihyperglycemic drugs be discontinued on the day of surgery. The guidelines also provide detailed advice on other choices, as previously mentioned^11^ (Table 8).

## Conclusion

Similarities and differences in the management of preoperative care for DM were identified across the four prominent organizations studied. These guidelines are based predominantly on expert opinion and consensus from best practice panels, resulting in different clinical practices globally. Differing recommendations highlight the lack of accessible data on perioperative DM care and the use of old guidelines, even though there is consensus on key elements. To enhance the quality of treatment for patients with DM undergoing surgical procedures, it is imperative to prioritize further research in this domain in the future. Furthermore, it promotes global collaboration in developing more consistent guidelines, enhancing patient results, and establishing similar clinical protocols in different healthcare environments.

### Competing interests

The authors have no conflicts of interest to declare.

## Figures and Tables

**Table 1.** Summary of target HbA1c for elective surgical procedures.

**HMC-Qatar guidelines**	**AoA guidelines**	**ADA guidelines!**	**ADS-ANZCA guidelines**

• Target HbA1c for elective surgery is ≤ 8.5%. • If HbA1c < 8.5% is not possible, then target the preoperative BGL between 5.6 and 12 mmol/L. • If HbA1c is >8.5%, admit to the hospital one to two days before the optimization of BGL.	• If HbA1c is >8.5%, delay elective surgery and refer to optimization.	• HbA1c levels should be maintained at < 8% for elective surgery whenever possible.	• Consider delaying surgery if HbA1c levels are >9%.


HMC-Qatar: Hamad Medical corporation, AoA: Association of Anaesthetists, ADA: American Diabetes Association, ADS-ANZCA: Australian and New Zealand College of Anaesthetists guidelines in collaboration with the Australian Diabetes Society, HbA1c: hemoglobin A1c, BGL: blood glucose levels.

**Table 2.** Summary of insulin management in the preoperative setting.

**HMC-Qatar guidelines**	**AoA guidelines**	**ADA guidelines!**	**ADS-ANZCA guidelines**

• Patients on diet control with FBG levels 10-13.9 mmol/L: start GIK therapy and additional bolus insulin (rapid-acting).• Patients on OHAs incretins: start basal form of long-acting insulin (0.2 U/kg SC).• Patients on insulin: reduce total daily dose by 20-30% and give normally on the morning of surgery.• Patients with insulin pumps: lower the infusion rate by 20-30% if DM is well controlled.• For all patients requiring hospitalization of >48 hours: change all forms of insulin to SC basal-bolus insulin (half dose as basal once daily, remaining dose as three injections as bolus insulin with meals).• Patients on NPH insulin: give two-thirds of the current regimen dose as basal glargine once daily in the morning.• Patients on premixed insulin: half of the usual dose as glargine on the morning of the procedure.• Stop taking prandial insulin during fasting.	• Short- and intermediate-acting combination of insulin: – Two separate injections per day with a properly calculated dose. – Half is given as intermediate-acting insulin in the morning with an unchanged evening dose.• Patients receiving 3-5 insulin injections:• Basal-bolus is omitted (in the morning and at lunchtime) but maintained for the morning procedures.• Patients on premixed insulin in the morning should receive it while omitting the midday dose. • Afternoon surgical procedure: omit morning and lunchtime doses.	• There are no specific ADA guidelines for the preoperative management of patients.• IV insulin should be used in critical care environments to achieve the desired glycemic control. • SC rapid- or short-acting insulin should be taken before meals or every 4-6 hours if the patient is fasting or receiving continuous enteral/parenteral nutrition.	• Clear home instruction on the insulin regimen is recommended for the healthcare team.• Basal insulin: continue as usual or reduce by 20% if overnight hypoglycemia is feared. • Combination of short- and long-acting insulin: reduce the morning dose by 50% and omit the lunch dose. Evening dose is unchanged (if drinking and eating).• Monitor BGL closely, 1-2 hours at home rather than in the hospital.• Target BGL of 5-10 mmol/L.• Colonoscopy procedure: omit short-acting insulin if the patient is fasting; give intermediate-acting, premixed, or co-formulated insulin in the morning and omit it at lunchtime. Give basal insulin as usual.


HMC-Qatar: Hamad Medical corporation, AoA: Association of Anaesthetists, ADA: American Diabetes Association, ADS-ANZCA: Australian and New Zealand College of Anaesthetists guidelines in collaboration with the Australian Diabetes Society, FBG: fasting blood glucose, GIK: glucose-insulin-potassium, OHAs: oral hyperglycemic agents, SC: subcutaneous, DM: diabetes mellitus, NPH: neutral protamine Hagedorn, IV: intravenous, BGL: blood glucose levels.

**Table 3.** Summary of VRIII management in the preoperative setting.

**HMC-Qatar guidelines**	**AoA guidelines**	**ADA guidelines!**	**ADS-ANZCA guidelines**

• Indications: surgical procedures exceeding 2 hours, ICU patients, emergency surgeries, BGL unmanaged with GIK.	• Indications: skipping multiple meals, tbl1DM undergoing a surgical procedure without receiving background insulin, uncontrolled diabetes, or emergency surgical procedures.	• No dedicated recommendations	• Use of VRIII exclusively in the intraoperative phase


HMC-Qatar: Hamad Medical Corporation in Qatar, AoA: Association of Anaesthetists, ADA: American Diabetes Association, ADS-ANZCA: Australian and New Zealand College of Anaesthetists guidelines in collaboration with the Australian Diabetes Society, ICU: intensive care unit, BGL: blood glucose levels, GIK: glucose-insulin-potassium, tbl1DM: type 1 diabetes mellitus, VRIII: variable rate intravenous insulin infusion.

**Table 4.** Summary of biguanide (metformin) use in the preoperative setting.

HMC-Qatar guidelines	**AoA guidelines**	**ADA guidelines!**	**ADS-ANZCA guidelines**

• Stop biguanides on the day of the surgical procedure. • Stop >24 hours: 65 years old, liver disease, heart failure, or renal impairment. • Do not start for 48 hours if IV contrast was used or if the patient is not drinking and eating normally.	• Individuals undergoing morning/afternoon procedures without IV contrast: discontinue the drug one day before admission. • Individuals with renal impairment: discontinue the agent before the surgical procedure and consider switching to another agent.	• Discontinue metformin on the day of surgery.	• Individuals with impaired kidney function, BGL >12 mmol/L before admission, colonoscopy, or major surgery: discontinue the agent while maintaining BGL with another agent of choice.


HMC-Qatar: Hamad Medical Corporation in Qatar, AoA: Association of Anaesthetists, ADA: American Diabetes Association, ADS-ANZCA: Australian and New Zealand College of Anaesthetists guidelines in collaboration with the Australian Diabetes Society, IV: intravenous, BGL: blood glucose levels.

**Table 5.** Summary of SGLT-2i use in the preoperative setting.

HMC-Qatar guidelines	**AoA guidelines**	**ADA guidelines!**	**ADS-ANZCA guidelines**

• Stop the drug three days before the scheduled surgery.	• Do not take the drug on the day of surgery.	• Stop SGLT-2i three to four days before surgery to reduce the risk of DKA.	• Stop the drug two days before surgery. • For minor surgery or day-stay procedures, omit the morning dose of the drug.


HMC-Qatar: Hamad Medical Corporation in Qatar, AoA: Association of Anaesthetists, ADA: American Diabetes Association, ADS-ANZCA: Australian and New Zealand College of Anaesthetists guidelines in collaboration with the Australian Diabetes Society, SGLT-2i: sodium-glucose cotransporter-2 inhibitors, DKA: diabetic ketoacidosis.

**Table 6.** Summary of GLP-1 receptor agonist use in the preoperative setting.

**HMC-Qatar guidelines**	**AoA guidelines**	**ADA guidelines!**	**ADS-ANZCA guidelines**

• Stop the drug on the morning of surgery.	• Continue the drug as directed until the time of surgery, whether the surgery is in the morning or evening.	• Stop the drug on the morning of surgery.	• Stop the drug on the morning of surgery.


GLP-1: glucagon-like peptide-1, HMC-Qatar: Hamad Medical Corporation in Qatar, AoA: Association of Anaesthetists, ADA: American Diabetes Association, ADS-ANZCA: Australian and New Zealand College of Anaesthetists guidelines in collaboration with the Australian Diabetes Society.

**Table 7.** Summary of DPP4-i use in the preoperative setting.

HMC-Qatar guidelines	**AoA guidelines**	**ADA guidelines!**	**ADS-ANZCA guidelines**

• Stop the drug on the morning of surgery.	• Continue the drug as directed until the time of surgery, whether the surgery is in the morning or evening.	• Stop the drug on the morning of surgery	• Stop the drug on the morning of surgery.


DPP4-i: dipeptidyl peptidase-4 inhibitors, HMC-Qatar: Hamad Medical Corporation in Qatar, AoA: Association of Anaesthetists, ADA: American Diabetes Association, ADS-ANZCA: Australian and New Zealand College of Anaesthetists guidelines in collaboration with the Australian Diabetes Society.

**Table 8.** Summary of OHA use in the preoperative setting.

**HMC-Qatar guidelines**	**AoA guidelines**	**ADA guidelines!**	**ADS-ANZCA guidelines**

• Stop all other OHAs on the day of surgery and consider insulin therapy if HbA1c exceeds 8.0%.	• If NPO (morning of the surgical procedure): stop meglitinides and acarbose. • Stop sulfonylurea on the morning of surgery. • Continue meglitinides and acarbose (morning dose) if surgery is scheduled in the afternoon of the same day. • If VRIII is initiated, stop all the agents mentioned previously.	• Stop other OHAs; replace with half a dose of NPH or 75–80% of a long-acting analogue or insulin pump, basal insulin, based on the type of DM and clinical judgment.	• Stop all OHAs on the morning of surgery.


OHAs: oral hyperglycemic agents, HMC-Qatar: Hamad Medical Corporation in Qatar, AoA: Association of Anaesthetists, ADA: American Diabetes Association, ADS-ANZCA: Australian and New Zealand College of Anaesthetists guidelines in collaboration with the Australian Diabetes Society, HbA1c: hemoglobin A1c, NPO: nil per os, VRIII: variable rate intravenous insulin infusion, NPH: neutral protamine Hagedorn, DM: diabetes mellitus.
